# Maternity care providers' use of health literacy tools and perceptions of barriers and facilitators

**DOI:** 10.1016/j.pecinn.2026.100509

**Published:** 2026-10-20

**Authors:** Evi M.E. Vlassak, Judit K.J. Keulen, Rianneke de Ritter, Elina Miteniece, Marijke J.C. Hendrix, Marianne J. Nieuwenhuijze

**Affiliations:** a Research Centre for Midwifery Science, Zuyd University of Applied Sciences, Maastricht, the Netherlands; b Care and Public Health Research Institute (CAPHRI), Maastricht University, Maastricht, the Netherlands

**Keywords:** Health literacy, Tools, Implementation, Maternity care, Mixed-methods

## Abstract

**Objectives:**

To explore maternity care providers' use of health literacy tools (HL-tools) and identify facilitators and barriers for implementation.

**Methods:**

Maternity care providers (*n* = 37) completed an e-learning on HL and HL-tools, followed by questionnaires exploring implementation barriers and facilitators, perceived usefulness, and tool use (before, current use, and future intentions). Focus groups further contextualized questionnaire findings and provided in-depth insights into participants' experiences.

**Results:**

After the e-learning, participants reported using nearly all HL-tools more frequently than before and intended to use them in the future. Participants described various barriers and facilitators, both tool-related and contextual, influencing their use of HL-tools. This also confronted them with several dilemmas. First, participants wanted to offer parents understandable, concise, visually supported tools, but perceived some as overly instructive or simplistic. Second, participants aimed for tools with reliable content but were often unfamiliar with HL-tools. Time constraints and competing priorities hindered familiarization. Third, participants valued having multiple sources on the same topic because this could better fit the diverse needs of parents but hesitated to select tools for parents due to those varying needs. Lastly, several participants mentioned barriers or facilitators that influenced their own use of HL-tools and, through them, the use of HL-tools by colleagues, creating a collective implementation tension.

**Conclusion:**

While maternity care providers strive to provide reliable and understandable information, they face both tool-related and contextual barriers.

**Innovation:**

Although HL-tools support tailored communication, they remain underused in maternity care. This study identifies implementation dilemmas to inform strategies for improving uptake.

## Highlights

• Providers value clear, concise tools, but reject ones seen as too simplistic.
• Providers want reliable tools, yet unfamiliarity leave tools' reliability unclear.
• Time constraints and competing priorities hinder providers' use of tools.
• Providers hesitate to choose tools for parents but value multiple sources.
• Colleagues' use of HL-tools influences individual providers' implementation.

## 1 Introduction

Health literacy (HL) is defined by the World Health Organization as “The cognitive and social skills which determine the motivation and ability of individuals to gain access to, understand and use information in ways to promote and maintain good health” [1]. It is a key factor in health inequalities [2] and a significant public health challenge across Europe [3]. Research indicates that 27–48% of Europeans struggle to understand fundamental health-related information [3]. Limited health literacy (LHL) is linked to longer hospital stays, lower use of preventive services, more emergency consultations, adverse health outcomes, and less effective communication with healthcare providers [4], [5], [6], [7], [8].

Also in maternity care, LHL is associated with adverse maternal and neonatal health outcomes [9], [10], [11], [12], [13], [14], [15]. Pregnant woman's understanding of health-related information can directly affect the development of the infant during gestation and throughout the formative years [16]. During pregnancy, (expectant) parents acquire information from different sources [17], [18]. The (expectant) parents' level of HL combined with the source characteristics (e.g. brochures, prescription labels, and conversations) influences how information is encoded, stored, and retrieved, affecting comprehension and incorporation into existing knowledge and decision-making processes [19], [20]. Healthcare providers can influence the susceptibility of (expectant) parents with LHL for information by speaking slowly, using simple language and visual images, which can help them to encode, store, and retrieve health information [21].

To tailor information sources to (expectant) parents' HL, providers should first assess their HL. In a previous study, we developed the conversational tool CHAT-maternity-care [22]. This practical tool raises providers' awareness for HL and offers examples of questions and observations in four domains to help maternity care providers estimate (expectant) parents' HL [22], [23]. However, estimating their HL is only meaningful if communication and information provision are subsequently adjusted to the (expectant) parents' HL. Nevertheless, specific innovative communication strategies are not consistently used in practice [24]. Previous research indicates that half of the Dutch healthcare providers rarely use innovative communication methods or materials specifically developed for tailoring care to (expectant) parents with LHL (HL-tools) [25]. This might be because of providers' limited awareness of the concept of HL or their struggle to systematically recognize individual HL needs [25], [26], [27], [28], [29]. It could also be that HL-tools are insufficiently implemented leading to healthcare providers being unfamiliar with them. Studies on use of HL-tools in different healthcare settings have revealed obstacles, including health professionals' limited awareness, uncertainty about usage, and skepticism regarding effectiveness [7].

To our knowledge, no research has explored the maternity care providers' perspectives regarding the use of HL-tools. With the wide range of available HL-tools, scattered across platforms, it remains unclear which ones are used and what factors facilitate or hinder their use. This study aims to explore which HL-tools maternity care providers use to tailor care to (expectant) parents with LHL, and to examine the facilitators and barriers for implementation.

## 2 Methods

### 2.1 Design

In this mixed-methods study, we explored maternity care providers experiences with the use and implementation of HL-tools in the Netherlands. Questionnaires provided an initial overview of HL-tool use and preliminary insights into perceived barriers and facilitators, which informed the in-depth exploration during the focus groups. Data were collected in June and July 2023.

### 2.2 Introduction in HL and relevant HL-tools

Participants completed an e-learning module 6–8 weeks before data collection. The e-learning was delivered through Easygenerator and had an estimated completion time of approximately 2 h. It provided 1) background on HL and LHL including the definition of HL, factors influencing HL, and the prevalence and consequences of LHL; 2) information on identifying LHL in (expectant) parents with CHAT-maternity-care [22]; and 3) relevant HL-tools (Table 1).

**Table 1:** selected HL-tools which were provided in the e-learning.

HL-tools	Description	Examples	When to apply
Decision aids and cards	Decision aids and cards that outline the pros and cons of a particular choice. Some decision aids also include a value clarification element.	Choice cards for miscarriage, breech presentation, contraceptives or decision aid for pain management during childbirth	Before or during consultation
Informative videos	Informative videos on topics related to pregnancy, childbirth, or the postpartum period.	Video on prenatal screening tests, video on breastfeeding, video on shaken baby syndrome	Before or after consultation
Visual stories	Information booklets written at B1 level with supportive illustrations.	Illustrated story on help with quitting smoking, illustrated story on the heel prick test for the baby	Before or after consultation
Signal card with instructions for when to call.	Cards display signals indicating when to call regarding pregnancy, childbirth, or the postpartum period. These signals are supported by illustrations.	Signal card when to call in pregnancy, signal card when to call postpartum	Before, during or after consultation
Antenatal classes	General antenatal classes offered by external providers.		During pregnancy
Informative sessions at the midwifery practice or hospital	Informative sessions at the midwifery practice and/or hospital providing information about pregnancy, childbirth, and/or the postpartum period.		During pregnancy
Poster “3 good questions”	Poster designed to support shared decision-making through three questions: - What are my options? - What are possible benefits and risks of these options? - What does this mean in my situation?		Before consultation
Website with information for non-native speakers and people with low literacy	Websites with information about pregnancy, childbirth, and the postpartum period in various languages, sometimes with audio or visual support.	*ZANZU.nl*, *voorlichtingvertaald.nl*	Before or after consultation
Interventions to strengthen the social network	Initiatives to strengthen the social network of the (expectant) parent.	*buurtmama's, buurtgezinnen, moeders informeren moeders*	After consultation
Informative apps	Apps that provide information about pregnancy, childbirth, and/or the postpartum period.	*Slimmer Zwanger*, *ZWapp*+, *ZwangerHap*	During pregnancy
Pictograms or illustrations	Pictograms or illustrations to support the conversation on topics in pregnancy, childbirth, or the postpartum period.	*Talk2mama*, pictogram cards	During consultation
Adjusted communication	Adjustment of providers' communication with patients with limited health literacy.	No technical jargon, speak slowly, avoid figurative language, teach-back method	During consultation

The research team together with a panel of experts collected, evaluated, and selected relevant HL-tools for the e-learning. These tools were accessible in the Netherlands and were derived from (scientific) literature and websites of professional organizations including PHAROS (Dutch Centre of Expertise on Health Disparities), the KNOV (Royal Dutch Organization of Midwives), RIVM (National Institute for Public Health and the Environment), NVOG (Dutch Society of Obstetrics and Gynecology), NHG (Dutch College of General Practitioners), and the Netherlands Patients Federation (Table 1). The expert panel included a professor in HL and patient participation, an obstetrician, midwives, maternity care assistants and participants representing the KNOV, Youth Healthcare organization, RIVM and maternity care assistant agencies. Detailed information on the selected HL-tools (in Dutch) is available in Appendix A.

### 2.3 Setting and participants

Participants were community midwives and maternity care assistants in the Netherlands, where 90% of pregnant women receive prenatal care from community midwives [30], and nearly all receive postnatal care from maternity care assistants and community midwives. Women with low-risk pregnancies receive antenatal, intrapartum, and postnatal care from a community midwife within midwife-led primary care. They may choose to give birth at home, in a birth centre, or in a midwife-led hospital setting. Almost all women spend their postpartum period at home, where maternity care assistants observe maternal and infant health and wellbeing and provide practical and supportive care for the mother and infant during the first eight days after birth, in collaboration with and under the supervision of the community midwife. Although the pathways to becoming a maternity care assistant (vocational education) or a midwife (Bachelor of Science degree) may differ between individuals, a clear national standard is set by the Ministry of Health that professionals must meet when graduating and accessing the workforce.

Participants were recruited through convenience sampling via advertisements on the website and social media of Zuyd University, School of Midwifery, and by sending emails to midwifery care practices and maternity care assistant agencies in the South of the Netherlands. Initial participants were asked to forward the invitation to colleagues. The email and advertisements provided information on research objectives, relevance, practical implications, inclusion criteria, and contact details. Maternity care assistants were permitted to participate during working hours, while midwives received financial compensation for time spend participating in the e-learning and focus groups.

### 2.4 Ethics

According to the ethics board of Maastricht University Medical Centre this study did not require ethical approval (number 2022–3283). Participants gave written consent, ensuring anonymity and the option to withdraw anytime. Data were stored on a secure server accessible only to the research team.

### 2.5 Data collection

Data were collected using two short questionnaires and six online in-depth focus groups with different participants.

The questionnaires were developed by the research team specifically for this study, with items directly aligned with the study objectives. The initial items were developed by EV and subsequently reviewed by the research team. Items were revised where necessary to ensure clarity and comprehensibility.

Participants received the first questionnaire a week before the focus group. This questionnaire included questions on demographics and two open-ended questions asking participants to name at least three perceived facilitators and three perceived barriers for using HL-tools to tailor care to (expectant) parents with LHL. Prior to the focus groups, responses were reviewed by the research team to inform the focus group guide and further explore previously reported barriers and facilitators.

The second questionnaire was completed online just before the start of the focus group. It contained four closed-ended (yes/no) questions about the twelve categories of HL-tools mentioned in Table 1: 1) Do you think this category of HL-tools is useful? 2) Did you use this category of HL-tools before completing the e-learning? 3) Do you use the category of HL-tools now? 4) Will you use the category of HL-tools in the future?

During the focus groups, we used a semi-structured focus group guide to explore perceived implementation barriers and facilitators of HL-tools. The following topics were discussed: 1) reasons for (not) using the category of HL-tools by care providers, informed by responses from the second questionnaire, 2) desired characteristics of HL-tools suitable for parents with LHL, 3) preferences regarding source, format, scope, and lay-out of HL-tools, 4) perceived availability and sufficiency of HL-tools for parents and care providers, and 5) experiences with and reasons for (not) using communication strategies to tailor care to HL. EV conducted all focus groups with alternating support from JK, EM, RR. All sessions were audio recorded, and field notes were taken to support reflection and interpretation during analysis.

### 2.6 Analysis

Responses to open-ended questions were analyzed per question using inductive coding (EV, EM). Closed question data were analyzed using descriptive statistics in SPSS version 29. Focus group recordings were transcribed verbatim and analyzed using inductive content analysis in NVivo version 11. During the initial coding round, codes were derived from the transcripts and merged where appropriate (EV). After establishing the code tree, transcripts were re-examined and recoded if needed (EV). JK, EM and four midwifery students checked the coding independently. Disagreements were discussed by EV, JK, EM until consensus was reached. A next level analysis synthesizing all findings deepened understanding of the dilemmas that emerged. Quotes from focus groups were translated into English to illustrate the findings.

### 2.7 Rigor and reflectivity

The mixed-methods research design ensured method triangulation of data collection [31]. Investigator triangulation was ensured through multiple researchers analyzing the data [31]. All research team members were experienced in qualitative research and had a background in midwifery and/or health sciences. Member-checks were conducted at the end of each focus group, where the interviewer summarized the discussion for participant confirmation, validating interpretations and ensuring accurate representation of perspectives. Data saturation appeared after the first four focus groups. The coding of the last two focus groups added no new codes. COREQ and GRAMMS guided the writing [32], [33].

## 3 Results

### 3.1 Background information and participant characteristics

Fourteen maternity care assistants (age 24–59 years, mean age 48.6 years, work experience 1–36 years) and 23 midwives (age 23–57 years, mean age 33.8 years, work experience 0.5–32 years) completed the e-learning and the two open-ended questions of the first questionnaire. Among the midwives, one worked in a solo practice, three in a duo practice, and nineteen in a group practice. The maternity care assistants and midwives were working in four of the twelve provinces of the Netherlands. Analysis of the two open-ended questions identified two primary codes (perceived barriers and facilitators) and 17 secondary codes, covering eight perceived barriers and nine perceived facilitators (Appendix B).

Six mixed focus groups were conducted, comprising 5–7 midwives and maternity care assistants per group (Appendix C). In total, 34 participants participated after one maternity care assistant and two midwives withdrew due to time constraints. The second questionnaire was completed just before the focus group by all midwives (*n* = 21) and 11 of 13 maternity care assistants. Two maternity care assistants did not complete the questionnaire due to technical issues. Focus groups lasted 34–43 min. Analyses of the focus groups identified two primary codes (perceived barriers and facilitators), each with eight secondary codes. Six tertiary codes were distributed across two secondary codes within perceived facilitators (Appendix D).

### 3.2 HL-tool use

Participants reported more frequent use of nearly all categories of HL-tools in the 6–8 weeks after completing the e-learning compared with before, except for antenatal classes and informational sessions, where usage was reported to remain the same or to decrease respectively (Fig. 1). Informative videos and adjusted communication were used most in the 6–8 weeks after completing the e-learning, while the “3 Good Questions” was used the least. The largest increases in use were reported for informative videos, visual stories, websites with information for non-native speakers and people with low literacy, and pictograms. Additionally, participants intended to use all categories of HL-tools equally or more in the future compared to the use in the 6–8 weeks after completing the e-learning.

**Fig. 1:**
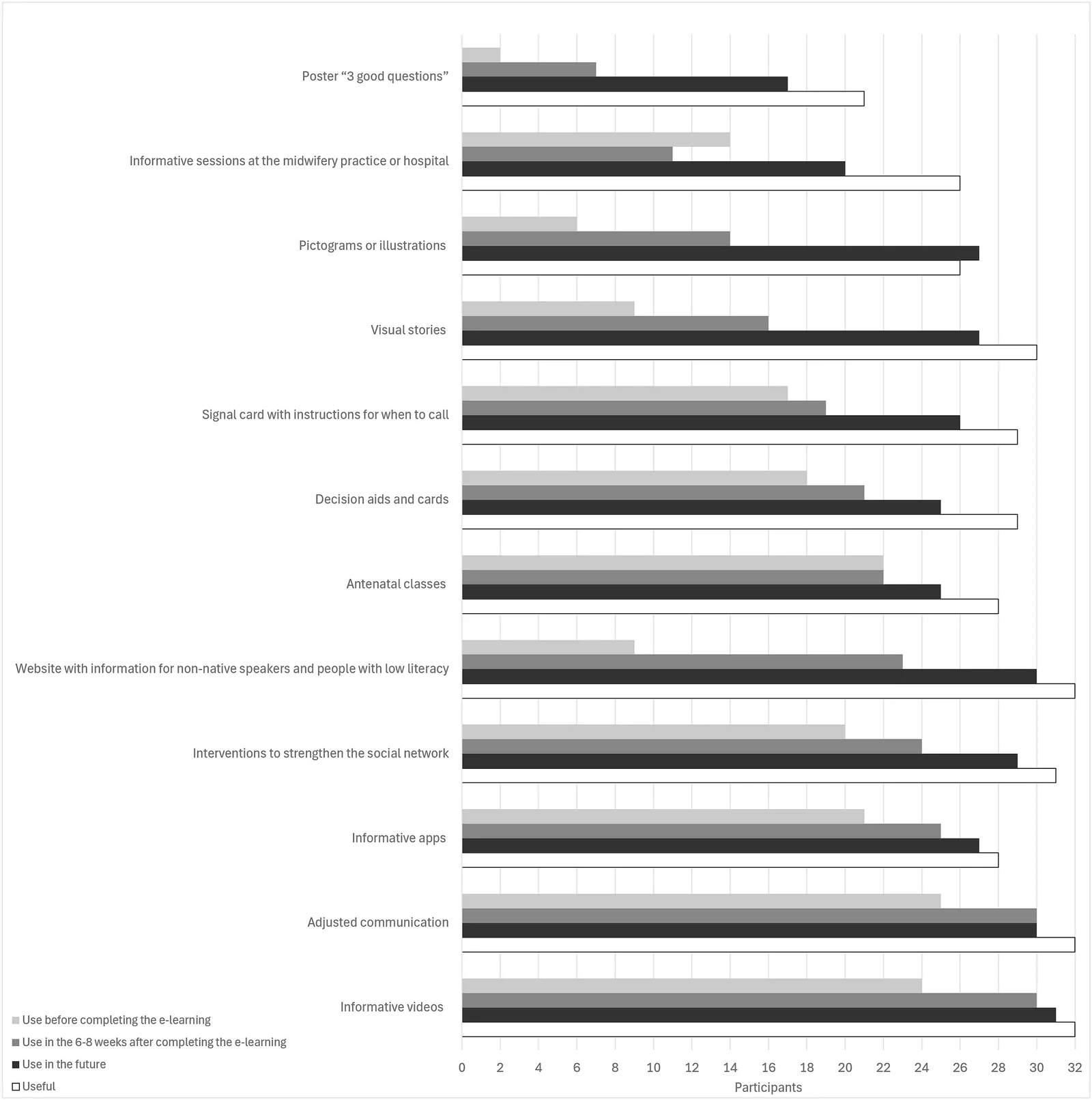
Perceived usefulness and usage of HL-tools by participants.

### 3.3 Perceived facilitators in HL-tool use

Participants appreciated tools that were easily accessible and user-friendly. They expressed different preferences for digital or paper availability of materials. Some found it helpful if they could easily share materials with (expectant) parents via email. Additionally, having tools available in one centralized location (i.e. the e-learning of this project) was perceived as beneficial. They said this accessibility allowed them to find the appropriate tools quickly and easily. Another facilitator mentioned was when HL-tools did not take much time to use, or when there was sufficient time available to use them. Furthermore, participants expressed the importance of tools providing reliable information.

“I think what is decisive for me is: Where does the information come from? The brochures from KNOV or RIVM, I know that information in those is accurate and reliable.” Midwife-p31

Participants said clarity and understandability of the tools facilitated their use. Participants preferred tools that conveyed information concisely in plain language. They mentioned that concise tools helped in communicating the message effectively and were appreciated by many, not only by (expectant) parents with LHL. Short sentences and paragraphs, avoiding technical jargon (or explanation if jargon is used), and images to support text were helpful in tailoring care to (expectant) parents with LHL. Additionally, having tools available in multiple foreign languages helped to overcome language barriers and ensured the correct information was conveyed.

“I also believe that keeping it short and concise is something everyone appreciates.” maternity care assistant-p24

Participants also expressed a preference for using visual materials, such as videos and pictograms instead of folders with plain text, to tailor care to (expectant) parents with LHL. Participants believed supportive visuals could aid in understanding text fragments.

“If you show the video of what happens when a baby does not latch properly, it brings a lot more awareness to parents like: ‘okay, it is really important that a baby latches correctly’. Then they have also seen what happens when it is not done properly.” maternity care assistant-P17

Participants appreciated having multiple tools addressing the same topic. Some said they believed repetition of the topic across different sources helped (expectant) parents to better retain the information. Variety allowed care providers and (expectant) parents to choose the source that best suited the (expectant) parents' needs. Participants were more likely to use a tool when their direct colleagues were also using it and when the tool addressed the needs of (expectant) parents. Participants perceived tools that could support verbal information as a facilitator for using that specific tool.

“But also the combination, right? People read and see something, then they also remember it better, so I think that combination is also good.” Midwife-p1

### 3.4 Perceived barriers in HL-tool use

Several factors hindered participants from using HL-tools. They mentioned that tailoring care to (expectant) parents with LHL was challenging when they were unfamiliar with the content of the tool, unaware of its existence, or unsure where to access it.

“What struck me was how much visual material is actually available for giving information. I really had no idea about some of the videos” midwife-p21

Participants found it challenging when their colleagues were unaware of or did not use HL-tools. They said that if they were the only ones in the practice or organization familiar with these HL-tools, they would likely not integrate them into their standard care. Tools not meeting the needs of (expectant) parents, parental reluctance to engage with HL-tools, and language barriers were also identified as barriers. Participants found it challenging and undesirable that they, as care providers, selected tools to offer to (expectant) parents. They said that preferences for tools were personal and varied widely.

“I think it would be more comfortable for (expectant) parents if they could choose what suits them, instead of us making that choice for them. That is, of course, very personal.” Midwife-p33

Participants also mentioned barriers such as time constraints, prioritizing other tasks, and lack of funding to support longer consultations. They said that tools requiring more time were used less frequently. Furthermore, participants mentioned that tools they experienced as user-unfriendly hindered use. For example, having videos without accompanying text was seen as problematic because they required extra explanation, discouraging their use in tailoring care to (expectant) parents with LHL.

“Some methods simply require more effort from the healthcare provider and also more time, which can sometimes make you deploy them less quickly.” Midwife-p9

Excessive length or complexity of the content of HL-tools was also mentioned as a barrier. Examples included extensive informational brochures, lengthy videos, and numerical risk information.

“I think the key is to provide the right information with as little information as possible. Because such a booklet is actually an overwhelming volume for many people.” Midwife-p13

Another perceived barrier was that some HL-tools were considered overly instructive, overly simplistic, or even infantilizing (Table 2).

“It is often difficult, for example, with the teach-back method: "I want to know if I explained it well, can you tell me what I said?" I find it quite a step to ask something like that. I feel it is too instructive. It is a very good method, but I find it difficult to use.” Midwife-p18

**Table 2:** Overview of the barriers and facilitators of using HL-tools as perceived by midwives and maternity care assistants in the Netherlands.

**Tool-related facilitators**	**Tool-related barriers**
Accessibility and user friendliness of tools^1^, ^2^	Not user-friendly^2^
Clear and understandable tools^1^	Perceived to be instructive, too simple, childish^2^
Short and concise tools^1^, ^2^	Too long or too complex^1^, ^2^
The tool was available in different languages^1^, ^2^ and used easy plain language^2^	
Reliable content in tools^1^, ^2^	
Visual materials^1^, ^2^	
Tool supports verbal communication^2^	
Different sources addressing the same topic^1^, ^2^	

**Contextual facilitators**	**Contextual barriers**
Enough time or using the tool did not take a lot of time^1^	Lack of time^1^, ^2^
Colleagues did also use the tool^2^	Colleagues did not use the tool^1^, ^2^
Tools met (expectant) parents' needs^1^	The tool did not meet (expectant) parents' needs or (expectant) parents did not want to cooperate when using HL-tools^1^
	Language barriers^1^
	Maternity care provider was not familiar with the content of the tool, unaware of its existence, or did not know where to find it^1^, ^2^
	Finding it undesirable that maternity care providers chooses tools for the (expectant) parent.^2^
	Finances^1^, ^2^
	Other priorities than using HL-tools^1^

1 Data from questionnaire.

2 Data from focus groups.

### 3.5 Identified dilemmas

While synthesizing the findings of the questionnaires and focus groups on perceived facilitators and barriers among participants using HL-tools, several dilemmas emerged. These dilemmas involved competing considerations that contributed to indecision among individual providers in using HL-tools. Additionally, a collective implementation tension also came forward.

#### 3.5.1 Clear but not too simplistic HL-tools

On the one hand, participants wanted to offer clear, concise, understandable, and visually supported tools to (expectant) parents; on the other hand, these tools were sometimes perceived as too simplistic, overly instructive, or childish by participants, which made providers uncomfortable and raised concerns about offending (expectant) parents when using these tools.

#### 3.5.2 Wanting to provide reliable tools but participants are unfamiliar with the tools and lacking time and priority to explore them

On the one hand, participants wanted to provide reliable content; on the other hand, participants were unaware of the existence of some tools and if they were aware of the existence they were not always familiar with the content of the HL-tool or did not know where to find it. Familiarizing themselves with the unknown tools required time and deliberate prioritization, while time constraints and competing priorities already posed a barrier to implementing these HL-tools.

#### 3.5.3 Valuing having multiple resources, yet hesitancy to select tools for parents

On the one hand, participants valued having multiple sources on the same topic because this accommodates parents' diverse needs; on the other hand, the variability of those needs fostered participants' hesitancy to recommend specific tools, leading them to prefer leaving the choice to parents themselves. This, together with their unfamiliarity with the tools, made it harder to ensure that parents found suitable and reliable information.

#### 3.5.4 Collective implementation tension

When a provider experienced barriers, this discouraged their own use of an HL-tool and, in the slipstream, colleagues' use. Conversely, if a colleague experienced facilitators and integrated a tool into practice, it could catalyze colleagues, making them more likely to adopt it. The adoption process is cumulative and reciprocal: one provider's decision to use or not use the tool could lower or raise the threshold for their colleagues to do the same, and vice versa, ultimately shaping how fully the tool becomes embedded in routine practice.

## 4 Discussion and conclusion

### 4.1 Discussion

This study explored maternity care providers' use of HL-tools to tailor care to (expectant) parents with LHL and identified perceived facilitators and barriers. Participants reported using nearly all twelve selected categories of HL-tools more frequently in the 6–8 weeks after completing the e-learning than before and intended to use them equally or more in the future. Participants described various tool-related and contextual barriers and facilitators influencing their use of HL-tools. This confronted them with several dilemmas. First, participants wanted to offer parents understandable, concise, visually supported tools, but perceived some as overly instructive or simplistic. Second, participants aimed to provide reliable content but often lacked familiarity with HL-tools, hindering assessment of their reliability and recommending them. Third, participants valued having multiple sources on the same topic because this addressed the diverse needs of parents but hesitated to select tools for parents due to those varying needs. Lastly, several participants mentioned barriers or facilitators that influenced their own use of HL-tools and, through them, the use of HL-tools by colleagues, creating a collective implementation tension.

Post e-learning, participating providers reported increased use and future intent to use HL-tools, which may suggest prior unawareness or uncertainty regarding their access and application. This aligns with a barrier identified in this study: unfamiliarity with the tool and its content. Education about HL and HL-tools, like our e-learning, can enhance awareness, knowledge, and confidence, thereby promoting adoption and implementation [34]. Moreover, consistent with previous research [35], centralizing HL-tools into one comprehensive database simplifies provider access and supports wider dissemination. However, raising awareness and simplifying access may still be insufficient for sustainable implementation. Other practical factors, like time constraints, limited funding to support longer consultations, and competing priorities, also hindered HL-tool use. This is consistent with previous research showing that limited resources and not prioritizing HL hinder providers' use of HL-specific knowledge and skills [36]. Selecting the right tool for the right person may help to use the available time as effectively as possible. To support providers in making this selection, instructions specifying the target population for which they were designed and proven effective could be added to the tools. The current findings also suggest that prioritizing HL is essential for structural changes that enable providers to spend enough time with each patient, not just in quantity, but tailored to the individual's needs. Prioritization of HL-tools is not only beneficial on the individual level, because the use of HL-tools was found to be peer-dependent: colleague use of HL-tools facilitated individual use, and non-use hindered it. Additionally, system-level prioritization of HL-sensitive care by government, policymakers, and funders can address time and funding constraints and support structural changes in workflow.

In the current study, providers preferred concise, easy-to-understand tools, that use plain language, and/or were visually supported, while they viewed lengthy, complex tools as less suitable when tailoring care to (expectant) parents with LHL. This aligns with research indicating that combining visual images with plain language can enhance understanding during pregnancy [37]. However, in this study, maternity care providers avoided HL-tools they perceived as too childish or simplistic because they felt uncomfortable using them and were concerned about offending (expectant) parents. This highlights the need for a delicate balance between clear but not too simplistic information. Previous research on visual decision aids suggests patient preferences vary significantly. While patients with sufficient HL considered visual decision aids overly simplistic and desired more background information, those with LHL still struggled with the provided text, underscoring the need to balance readability with content integrity to support informed decision-making [38], [39]. Additionally, research has shown that women with lower levels of education use written information sources, like leaflets and websites, less frequently than women with middle and higher levels of education [18]. Given these differences in information needs, it is essential for providers to estimate (expectant) parents' HL and to have different resources available to tailor information effectively.

Furthermore, providers emphasized the importance of having reliable tools and multiple sources addressing the same topic, ensuring a variety of trustworthy options aligning with parents' needs. However, they also expressed reluctance to select tools on behalf of (expectant) parents because they valued the parents' personal choice. Previous research indicates it is important for (expectant) parents to find or receive information that aligns with their needs: the right amount of information at the appropriate time in pregnancy [40]. Other previous research shows patients in maternity care seek information from various sources [18], [41], yet they consider the guidance from midwives and obstetricians the most useful and trustworthy [18]. Women with low basic skills and primigravida struggle with finding reliable information, understanding probabilistic information, and applying it to their own situation to shape preferences [42]. This suggests that, despite their hesitation, providers would be better off selecting (together with (expectant) parents) and/or guiding them to tools rather than leaving (expectant) parents to search or choose on their own, as this could ensure parents access to reliable and relevant information and perceive this information as more trustworthy and useful.

#### 4.1.1 Strengths and limitations

A strength of this study is its data collection design, which combines an exploratory initial questionnaire with in-depth focus groups. Although the quantitative component included a small sample and provided descriptive results only, it offered an initial overview of HL-tool use and informed the in-depth exploration during the focus groups. Furthermore, to our knowledge, this is the first study to explore why maternity care providers do or do not use HL-tools to tailor care to the needs of (expectant) parents. The tools provided in the e-learning were carefully selected with the research team and an expert panel, ensuring a comprehensive and well-founded representation of currently available HL-tools for maternity care in the Netherlands.

A limitation is the potential for selection bias. Providers already engaged in tailoring care to HL may have been more likely to participate, possibly leading to an overrepresentation of tool use and perceived usefulness. This may also have influenced the reported barriers and facilitators, as providers with greater HL awareness or experience may perceive fewer barriers to implementation than those with less familiarity with HL. However, implementation often begins with innovators and early adopters who are willing to test and integrate novel approaches and could thereby provide valuable insights into practical barriers and facilitators that can guide broader implementation [43]. The inclusion of participants from different regions, ages, professional roles, practice settings, and career stages contributed to a range of perspectives on the implementation of HL-tools in the Netherlands. Educational qualifications were not collected, both professions have a defined national training route, but variation in additional training could therefore not be examined. Furthermore, the reported increase in the use of HL-tools was based on retrospective self-report and may therefore be subject to recall bias, potentially resulting in over- or underestimation of previous tool use. Although a pre-post intervention assessment would have provided a stronger basis for comparison, these findings remain valuable as they provide exploratory insights into participants' perceived changes in the use of HL-tools. In addition, some categories of HL-tools included multiple specific tools addressing different topics. Therefore, participants' responses regarding use and perceived usefulness may reflect their overall perception of the tool category rather than each individual tool. Nevertheless, assessing tool categories allowed us to obtain a broader overview of perceived usefulness and implementation considerations while limiting participant burden. Generalizability to other countries is limited due to the usage of Dutch HL-tools. Nevertheless, the insights highlight key factors influencing implementation of HL-tools in maternity care, which are also relevant for other (maternity) healthcare contexts where providers aim to improve communication and care for individuals with LHL through HL-tools.

### 4.2 Innovation

Although communication strategies and supporting tools are available to help providers tailor information to people with LHL [44], and HL interventions have been studied for pregnant women with limited language proficiency [45], the integration of HL practices into routine maternity care remains limited [25], [29]. The novelty of this study lies in examining multiple categories of HL-tools simultaneously, among both midwives and maternity care assistants in primary maternity care, and in identifying the competing considerations that leave providers undecided about using them. Previous research in another healthcare setting has examined providers' perceived barriers and facilitators related to the implementation of HL practices [46]. While that study identified several recurring factors influencing implementation (e.g. time and workflow constraints), the present study shows how such factors play out across a range of tool categories in primary maternity care. These insights can inform the development of implementation strategies for HL-tools in maternity care practice. This might include the development of a centralized database, a balance between clear but not too simplistic information, collective priority setting, and training for professionals in estimating parents' HL followed by using suitable HL-tools.

### 4.3 Conclusion

Maternity care providers encounter both facilitators and barriers using HL-tools, that together created several dilemmas. While they strive to provide reliable and understandable information, they face tool-related and contextual barriers.

## Statement

The manuscript is our original work and only submitted to *PEC Innovation*. It has not been published in any journal or other citable form.

## CRediT authorship contribution statement

**Evi M.E. Vlassak:** Writing – review & editing, Writing – original draft, Visualization, Validation, Project administration, Methodology, Investigation, Formal analysis, Data curation, Conceptualization. **Judit K.J. Keulen:** Writing – review & editing, Validation, Project administration, Methodology, Investigation, Formal analysis, Data curation, Conceptualization. **Rianneke de Ritter:** Writing – review & editing, Validation, Methodology, Investigation, Conceptualization. **Elina Miteniece:** Writing – review & editing, Validation, Investigation. **Marijke J.C. Hendrix:** Writing – review & editing, Supervision, Project administration, Methodology, Conceptualization. **Marianne J. Nieuwenhuijze:** Writing – review & editing, Supervision, Methodology, Funding acquisition, Conceptualization.

## Ethics declaration

Written informed consent to take part in the study and to publish the article has been obtained from all participants or their legal representatives. The privacy rights of participants have been observed.

This study was performed in compliance with relevant laws, regulatory frameworks and guidelines where the research took place. The ethics board of Maastricht University Medical Centre granted an exemption. The authors provided the following explanation: According to the ethics board of Maastricht University Medical Centre this study is not subject to the Medical Research Involving Human Subjects Act (WMO) and did not require ethical approval (number 2022-3283).

## Declaration of generative AI and AI-assisted technologies in the writing process

During the preparation of this work the authors used ChatGPT-5 in order to improve the linguistic quality of the manuscript, since the authors are not native English speakers. After using this tool/service, the authors reviewed and edited the content as needed and take full responsibility for the content of the published article.

## Funding

This work was supported by the 10.13039/501100010409Nationaal Regieorgaan Praktijkgericht Onderzoek SIA (Regieorgaan SIA) under Grant RAAK.PUB08.004. The funding body has no role in the conceptualization, design, data collection, analysis, decision to publish and/or preparation of the manuscript.

## Declaration of competing interest

The authors declare that they have no known competing financial interests or personal relationships that could have appeared to influence the work reported in this paper.

## Figures and Tables

**Table:** 

HL-hulpmiddelen	Hulpmiddelen geïncludeerd in de e-learning binnen deze groep	Bron
Keuzehulpen en -kaarten	Keuzekaarten (Anticonceptie, Miskraam, Zwangerschap buiten de baarmoeder, Bevallen nare bevallingservaring, GBS-bacterie, Stuitligging, Zwanger 39 weken, Zwanger 41 weken)	Thuisarts
	Keuzehulp: Omgaan met pijn tijdens de bevalling	Thuisarts
Informatieve video's	Een baby krijgen en hoe werkt dat in Nederland	IJmond geboortezorg
	Een baby krijgen en organisaties die kunnen helpen	IJmond geboortezorg
	Een baby krijgen en leefstijl, voeding en werk	IJmond geboortezorg
	Een baby krijgen en wat er allemaal verandert	IJmond geboortezorg
	Een baby krijgen en stress hebben	IJmond geboortezorg
	Zwanger in Nederland	KNOV
	Gezond Zwanger Worden	KNOV
	Gezond Zwanger Zijn	KNOV
	Niet vitale zwangerschap en miskraam	KNOV/NVOG
	Bloedonderzoek voor Zwangeren	RIVM
	NIPT	RIVM
	13 wekenecho en 20 wekenecho	RIVM
	Vaccinatie en zwanger	NVOG
	Mogelijkheden als je 41 weken zwanger bent	KNOV/NVOG
	Inleiden van de bevalling	KNOV/NVOG
	Wanneer bellen?	KNOV
	Omgaan met weeën	KNOV
	Pijnbestrijding in het ziekenhuis	KNOV
	Kraamzorg	KNOV
	Kraamzorg	BO geboortezorg/Pharos
	Shaken baby	Kraamzorgbureau
	De hielprik bij ouders thuis	RIVM
	Gehoortest	RIVM
	Borstvoeding	Voedingscentrum
	Flesvoeding	Voedingscentrum
	Veilig slapen	Veiligheid NL
Beeldverhalen	Het beste moment om zwanger te worden	Nederlands Jeugdinstituut (momenteel niet meer op website)
	Straks Zwanger Worden	www.strakszwangerworden.nl (CPZ)
	Bloedonderzoek	RIVM
	In contact met je kindje	Pharos
	Hulp bij stoppen met roken	Pharos
	Rookvrij blijven doe je met hulp	Pharos
	Kraamzorg	Pharos
	Hielprik	RIVM
	Gehoortest	RIVM
	Wat kun je doen als je baby huilt	Pharos
	Daphne is gek op haar baby en op haar telefoon	Pharos
	Meisjes Besnijdenis: wat kan Sara doen?	Pharos (momenteel niet meer op website)
	Borstvoeding	Nederlands Jeugdinstituut (momenteel niet meer op website)
	Slapen	Nederlands Jeugdinstituut(momenteel niet meer op website)
Signaalkaarten met instructies wanneer contact op te nemen	Signaalkaart Zwangerschap	KNOV (momenteel niet meer op website)
	Signaalkaart Net Bevallen	KNOV (momenteel niet meer op website)
Zwangerschapscursussen	Zwangerschapscursussen aangeboden door externe aanbieders.	–
Voorlichtingsavonden in de verloskundigenpraktijk of het ziekenhuis	Informatieve sessies in de verloskundigenpraktijk en/of het ziekenhuis waarin informatie wordt gegeven over zwangerschap, bevalling en/of de kraamperiode.	–
Poster “3 goede vragen”	Zwanger – 3 goede vragen	www.3goedevragen.nl
Website met informatie voor anderstalige en mensen die laaggeletterd zijn	Gezinsplanning, anticonceptie en zwangerschap — Zanzu	www.zanzu.nl
	www.voorlichtingvertaald.nl	www.voorlichtingvertaald.nl
Interventies om het sociale network te versterken	Buurtmama's	Buurtmama's
	Moeders informeren moeders	Moeders informeren moeders
	Buurtgezinnen	Buurtgezinnen
	Steunouder	Steunouder
	Eigen Kracht	Eigen Kracht
	Mamacafe	–
Informatieve apps	ZwApp+	ZwApp+
	Slimmer Zwanger	Erasmus MC
	Babybalance	Babybalance
	ZwangerHap	Voedingscentrum
Pictogrammen of illustraties	Pictogramkaart Zwangerschap	Zuyd Hogeschool
	Pictogramkaart Kraambed	Zuyd Hogeschool
	Talk2mama	Talk2mama
Aangepaste communicatie	Aanpassing van de communicatie door zorgverleners met patiënten met beperkte gezondheidsvaardigheden: geen vakjargon gebruiken, langzaam spreken, beeldspraak vermijden en de terugvraagmethode (teach-back) toepassen. Hiervoor kregen zorgverleners informatie via: Checklist communicatie op maat (ZANGG) De terugvraagmethode: infosheet | Pharos Gesprekstechnieken in de zorg - YouTube	Zuyd Hogeschool, Pharos

**Table:** 

Primary codes	Secondary codes
Perceived facilitators	Enough time or using the tool did not take a lot of time.
	Clear and understandable tools
	Different sources addressing the same topic
	Accessibility and user friendliness of tools
	Short and concise tools
	The tool was available in different languages
	Reliable content in tools
	Tools met (expectant) parents' needs
	Visual materials
Perceived barriers	Time
	Finances
	The tool did not meet (expectant) parents' needs or (expectant) parents did not want to cooperate when using HL-tools
	Maternity care provider was not familiar with the content of the tool, unaware of its existence, or did not know where to find it
	The tool was too long or too complex
	Colleagues did not use the tool
	Language barriers
	No priority to use HL-tools

**Table:** 

Focus group	Midwives (n)	Maternity care assistant (n)	Total participants (n)
Focus group 1	1	4	5
Focus group 2	4	3	7
Focus group 3	3	2	5
Focus group 4	5	2	7
Focus group 5	4	1	5
Focus group 6	4	1	5

**Table:** 

Primary codes	Secondary codes	Tertiary codes
Perceived facilitators	Visual materials	
	Short and concise tool	
	Reliable content in tools	
	Colleagues do also use tool	
	The tool supported verbal communication	
	Different sources addressing the same topic	
	Language	Easy plain language in tool
		The tool was available in different languages
	Accessibility and user friendliness of tools	Finding all tools in one place
		Digital
		On paper
		Sharing via mail
Perceived barriers	The tool was perceived to be instructive, too simple, childish	
	Colleagues did not use the tool	
	Finances	
	Time	
	Tool was not user-friendly	
	Tool was too long or too complex	
	Finding it undesirable that maternity care providers chooses tools for the (expectant) parent.	
	Maternity care provider was not familiar with the content of the tool, unaware of its existence, or did not know where to find it	

## Data Availability

The data is available from the corresponding author on reasonable requests due to privacy restrictions.

## References

[1] Nutbeam D. (1998).

[2] World Health Organization (2015).

[3] Baccolini V., Rosso A., Di Paolo C., Isonne C., Salerno C., Migliara G. (2021). What is the prevalence of low health literacy in European Union member states? A systematic review and meta-analysis. J Gen Intern Med.

[4] Schillinger D., Bindman A., Wang F., Stewart A., Piette J. (2004). Functional health literacy and the quality of physician-patient communication among diabetes patients. Patient Educ Couns.

[5] Cavanaugh K.L., Wingard R.L., Hakim R.M., Eden S., Shintani A., Wallston K.A. (2010). Low health literacy associates with increased mortality in ESRD. J Am Soc Nephrol.

[6] Fransen M.P., Stronks K., Essink-Bot M.L. (2011). Laaggeletterdheid te lijf.

[7] Heijmans M., Zwikker H., van der Heide I., Rademakers J. (2016).

[8] Berkman N.D., Donahue K., Halpern D., Sheridan S.L., Donahue K.E., Halpern D.J. (2011). Low health literacy and health outcomes: an updated systematic review. Ann Intern Med.

[9] Vila-Candel R., Navarro-Illana E., Mena-Tudela D., Pérez-Ros P., Castro-Sánchez E., Soriano-Vidal F.J. (2020). Influence of puerperal health literacy on tobacco use during pregnancy among Spanish women: a transversal study. Int J Environ Res Public Health.

[10] Lupattelli A., Picinardi M., Einarson A., Nordeng H. (2014). Health literacy and its association with perception of teratogenic risks and health behavior during pregnancy. Patient Educ Couns.

[11] Yee L.M., Silver R., Haas D.M., Parry S., Mercer B.M., Wing D.A. (2021). Association of health literacy among nulliparous individuals and maternal and neonatal outcomes. JAMA Netw Open.

[12] Yin H.S., Sanders L.M., Rothman R.L., Shustak R., Eden S.K., Shintani A. (2014). Parent health literacy and “obesogenic” feeding and physical activity-related infant care behaviors. J Pediatr.

[13] Poorman E., Gazmararian J., Elon L., Parker R. (2014). Is health literacy related to health behaviors and cell phone usage patterns among the text4baby target population?. Arch Public Health.

[14] Endres L.K., Sharp L.K., Haney E., Dooley S.L. (2004). Health literacy and pregnancy preparedness in pregestational diabetes. Diabetes Care.

[15] Kaufman H., Skipper B., Small L., Terry T., McGrew M. (2001). Effect of literacy on breast-feeding outcomes. South Med J.

[16] Ferguson B. (2008). Health literacy and health disparities: the role they play in maternal and child health. Nurs Womens Health.

[17] Song F.W., West J.E., Lundy L., Smith Dahmen N. (2012). Women, pregnancy, and health information online. Gend Soc.

[18] Vogels-Broeke M., Daemers D., Budé L., de Vries R., Nieuwenhuijze M. (2022). Sources of information used by women during pregnancy and the perceived quality. BMC Pregnancy Childbirth.

[19] Lang A. (2006). Using the limited capacity model of motivated mediated message processing to design effective cancer communication messages. J Commun.

[20] Squiers L., Peinado S., Berkman N., Boudewyns V., McCormack L. (2012). The health literacy skills framework. J Health Commun.

[21] Weiss B.D. (2007).

[22] Vlassak E.M.E., Miteniece E., Keulen J.K.J., Gravendeel M., Korstjens I., Budé L. (2024). Development of the Conversational Health Literacy Assessment Tool for maternity care (CHAT-maternity-care): participatory action research. BMC Health Serv Res.

[23] Vlassak E.M.E., Keulen J.K.J., Miteniece E., de Ritter R., Hendrix M.J.C., Nieuwenhuijze M.J. (2025). Care providers' and parents' experiences with implementing the Conversational Health Literacy Assessment Tool (CHAT)-maternity-care in the Netherlands: a mixed methods study. Healthcare (Basel).

[24] Mackert M., Ball J., Lopez N. (2011). Health literacy awareness training for healthcare workers: improving knowledge and intentions to use clear communication techniques. Patient Educ Couns.

[25] Murugesu L., Heijmans M., Rademakers J., Fransen M.P. (2022). Challenges and solutions in communication with patients with low health literacy: perspectives of healthcare providers. PloS One.

[26] Storms H., Aertgeerts B., Vandenabeele F., Claes N. (2019). General practitioners' predictions of their own patients' health literacy: a cross-sectional study in Belgium. BMJ Open.

[27] Ohl M., Harris A., Nurudtinova D., Cai X., Drohobyczer D., Overton E.T. (2010). Do brief screening questions or provider perception accurately identify persons with low health literacy in the HIV primary care setting?. AIDS Patient Care STDS.

[28] Dickens C., Lambert B.L., Cromwell T., Piano M.R. (2013). Nurse overestimation of patients' health literacy. J Health Commun.

[29] Creedy D.K., Gamble J., Boorman R., Allen J. (2021). Midwives' self-reported knowledge and skills to assess and promote maternal health literacy: a national cross-sectional survey. Women Birth.

[30] Perined (2020).

[31] Korstjens I., Moser A. (2018). Series: practical guidance to qualitative research. Part 4: trustworthiness and publishing. Eur J Gen Pract.

[32] Tong A., Sainsbury P., Craig J. (2007). Consolidated criteria for reporting qualitative research (COREQ): a 32-item checklist for interviews and focus groups. Int J Qual Health Care.

[33] O'Cathain A., Murphy E., Nicholl J. (2008). The quality of mixed methods studies in health services research. J Health Serv Res Policy.

[34] Gibson C., Smith D., Morrison A.K. (2022). Improving health literacy knowledge, behaviors, and confidence with interactive training. Health Lit Res Pract.

[35] Greaney M.L., Wallington S.F., Rampa S., Vigliotti V.S., Cummings C.A. (2020). Assessing health professionals' perception of health literacy in Rhode Island community health centers: a qualitative study. BMC Public Health.

[36] Connell L., Finn Y., Sixsmith J. (2023). Health literacy education programmes developed for qualified health professionals: a scoping review. BMJ Open.

[37] Vamos C.A., Merrell L., Detman L., Louis J., Daley E. (2019). Exploring women's experiences in accessing, understanding, appraising, and applying health information during pregnancy. J Midwifery Womens Health.

[38] Noordman J., Noordam D., van Treeck J., Prantl K., Pennings P., Borsje P. (2024). Visual decision aids to support communication and shared decision-making: how are they valued and used in practice?. PloS One.

[39] Naghdi M., Cao P., Essers R., Heijligers M., Paulussen A.D.C., van der Lugt A. (2025). Artificial intelligence-simplified information to advance reproductive genetic literacy and health equity. Hum Reprod.

[40] Baas C.I., Erwich J.J., Wiegers T.A., de Cock T.P., Hutton E.K. (2015). Women's suggestions for improving midwifery care in the Netherlands. Birth.

[41] Ghiasi A. (2021). Health information needs, sources of information, and barriers to accessing health information among pregnant women: a systematic review of research. J Matern Fetal Neonatal Med.

[42] Murugesu L., Damman O.C., Derksen M.E., Timmermans D.R.M., de Jonge A., Smets E.M.A. (2021). Women's participation in decision-making in maternity care: a qualitative exploration of clients' health literacy skills and needs for support. Int J Environ Res Public Health.

[43] Rogers E.M. (1995).

[44] Noordman J., van Vliet L., Kaunang M., van den Muijsenbergh M., Boland G., van Dulmen S. (2019). Towards appropriate information provision for and decision-making with patients with limited health literacy in hospital-based palliative care in Western countries: a scoping review into available communication strategies and tools for healthcare providers. BMC Palliat Care.

[45] Khan M., Dave A., Benton M., Moss N., Kaler M.K. (2024). Health literacy interventions for pregnant women with limited language proficiency in the country they live in: a systematic review. BMC Public Health.

[46] Howe C.J., Lewis B., Edmondson S. (2024). Barriers and facilitators to implementing health literacy practices in a pediatric ENT clinic: a mixed-methods study. J Nurs Care Qual.

